# Effects of DBS in parkinsonian patients depend on the structural integrity of frontal cortex

**DOI:** 10.1038/srep43571

**Published:** 2017-03-06

**Authors:** Muthuraman Muthuraman, Günther Deuschl, Nabin Koirala, Christian Riedel, Jens Volkmann, Sergiu Groppa

**Affiliations:** 1 Department of Neurology, University Medical Center of the Johannes Gutenberg University, Mainz, Germany; 2 Department of Neurology, Christian Albrechts University, Kiel, Germany; 3 Department of Neuroradiology, Christian Albrechts University, Kiel, Germany; 4 Department of Neurology, Julius Maximilians University, Würzburg, Germany

## Abstract

While deep brain stimulation of the subthalamic nucleus (STN-DBS) has evolved to an evidence-based standard treatment for Parkinson’s disease (PD), the targeted cerebral networks are poorly described and no objective predictors for the postoperative clinical response exist. To elucidate the systemic mechanisms of DBS, we analysed cerebral grey matter properties using cortical thickness measurements and addressed the dependence of structural integrity on clinical outcome. Thirty one patients with idiopathic PD without dementia (23 males, age: 63.4 ± 9.3, Hoehn and Yahr: 3.5 ± 0.8) were selected for DBS treatment. The patients underwent whole-brain preoperative T1 MR-Imaging at 3 T. Grey matter integrity was assessed by cortical thickness measurements with FreeSurfer. The clinical motor outcome markedly improved after STN-DBS in comparison to the preoperative condition. The cortical thickness of the frontal lobe (paracentral area and superior frontal region) predicted the clinical improvement after STN-DBS. Moreover, in patients with cortical atrophy of these areas a higher stimulation voltage was needed for an optimal clinical response. Our data suggest that the effects of STN-DBS in PD directly depend on frontal lobe grey matter integrity. Cortical atrophy of this region might represent a distinct predictor of a poor motor outcome after STN-DBS in PD patients.

Deep brain stimulation (DBS) of the subthalamic nucleus (STN) is an evidence based standard treatment procedure for Parkinson’s disease (PD)^1^. DBS of the STN can improve not only motor symptoms but also non-motor deficits and levodopa-induced motor complications in patients with PD, leading to a significant improvement of the overall quality of life. Recent research has raised three important questions for this highly innovative and promising therapeutic procedure: patient selection^2^, timing for DBS^3^ and optimal stimulation site^4^. Answers to the above questions may possibly be obtained by better understanding the pathophysiology of DBS effects, which are still unclear^5^. A complex modulation of the basal ganglia loops or the cortico-subcortical networks is hypothesised^6^,^7^. DBS presumably not only changes the neural activity in the nuclei but also targets fibre tracts entering, exiting, or passing the stimulation site^8^. Studies on primates and recent studies on humans have confirmed the existence of so-called hyperdirect cortical STN projections to the supplementary motor area (SMA) and primary motor cortex (M1)^9^, which might be important for the effects of STN-DBS. The direct connections of the STN to the frontal cortex and M1 were shown to essentially contribute to the therapeutic effects of STN-DBS in a rodent model of PD^10^, and STN-DBS directly modified the firing probability of the cortifugal projection neurons in M1 that resolved PD symptoms and improved motor control^11^. A modulation of the pathological oscillations in the frontal brain networks through the stimulation of the hyperdirect pathway might be achieved^12^. The aberrant cyto-architectural alterations of these cortical regions could play a key role for the modulation of pathological oscillations and be directly linked to differing patient responsiveness to STN-DBS. We hypothesize that the cortical integrity of frontal regions is a predictor of the DBS response. In this study, we applied preoperative cortical thickness measurements as a parameter of grey matter integrity and morphology. These measurements have the advantage of providing a direct quantitative index^13^ and are linked to the number of cells within a column, reflecting grey matter volume, density and the arrangement of neurons and neuropil in a biological and topological meaningful way. Previous studies associated cortical thinning with aging^14^ and mild cognitive impairment in PD^15^.

## Subjects and Methods

Thirty-one patients with idiopathic PD without dementia selected for DBS treatment (exclusion criteria: a score of ≤130 on the Mattis Dementia Rating scale) were included in this study (23 males, 8 females; age 63.4 ± 9.3; Hoehn and Yahr 3.5 ± 0.8 medication OFF, 2.6 ± 0.7 medication ON). The demographic details of the patients are listed in Table 1. The selected patients collectively underwent standardized pre- and postoperative assessment. None of the patients experienced complications; in particular, there were no cerebral bleedings. The local ethics committee (Medical faculty, University Clinic Schleswig Holstein, Kiel) approved the study protocol and all patients gave their informed written consent. All methods were performed in accordance with the relevant guidelines and regulations. All patients received, after clinical assessment, bilateral STN electrodes (see below). To assess changes in clinical outcome, the universal Parkinson disease rating scale (UPDRS_III_) motor score quotient was utilized, and was defined as


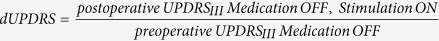


The defined Medication OFF (MED OFF) state was achieved when a patient had been OFF medication for at least 12 hours (slow release formations, dopamine agonist and longer lasting drugs were stopped for 72 hours). The assessment was performed at least one hour after waking in the morning to eliminate a possible “sleep benefit” and occurred after the administration of a dose of liquid levodopa that was 50 percent higher than the usual morning dose of dopaminergic medication.

### Surgical procedure and stimulation parameters

The surgical procedure has been previously described in detail^16^,^17^,^18^. Magnetic resonance imaging (MRI), and microelectrode recording were used for targeting STN. The permanent electrode (model 3389 DBS, Medtronic) and pulse generators (Activa) were implanted. Postoperatively, the optimal stimulation settings and dopaminergic medication were progressively adjusted. The pulse setting was in a monopolar setting at 60 μsec in duration at 130 Hz in all patients; voltage was adjusted for each individual patient. For the analysis, we considered the stimulation parameters at a stable state (Stimulation On (STIM ON), at least 3 months after implantation). The stimulation voltage (further as *DBS voltage*) necessary for the optimal clinical response was measured (in millivolts) at the active electrode. The voltage ranges in these patients for the left and right sides were 0.5 to 4.0 V (mean ± std: 2.16 ± 0.84 V) and 0.8 to 4.4 V (mean ± std: 2.21 ± 0.86 V), respectively.

### MRI data acquisition

All patients underwent a preoperative high resolution MRI, performed on a 3 T MR-Scanner (Philips Achieva) using an 8-channel SENSE head coil. We obtained a high-resolution T1-image of the brain using a magnetization-prepared rapid gradient echo (MPRAGE) sequence (Response time (TR) = 7.7 ms, Echo time (TE) = 3.6 ms, flip angle = 8°, 160 slices, slice thickness = 1 mm, matrix = 256 × 256 mm, isotropic resolution = 1 × 1 × 1 mm).

### Cortical thickness analysis

**Figure 1:**
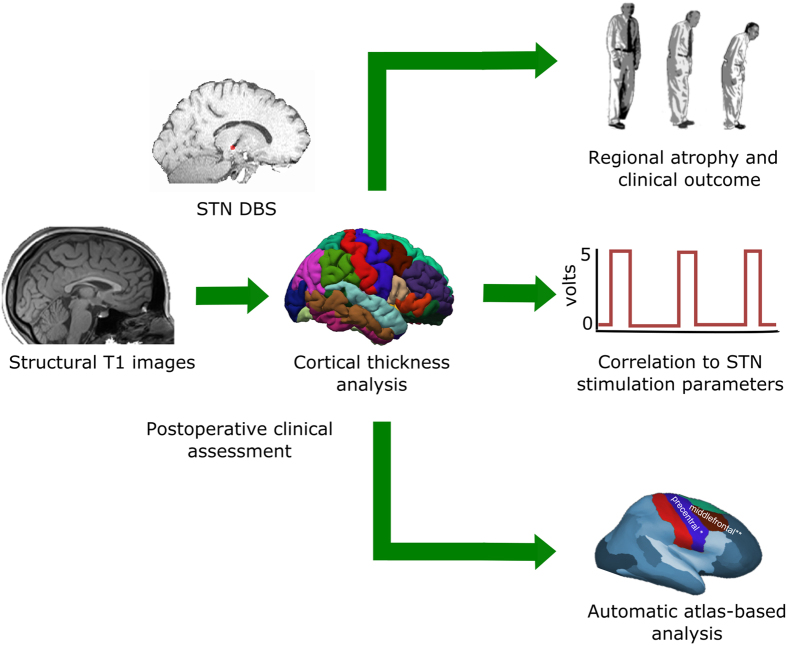
The study workflows of the analyses are depicted. Cortical thickness was estimated with 3D-T1 images and then following analyses were done, namely (1) correlation to the post-operative motor improvement, (2) regional atrophy and clinical outcome, and finally (3) automatic atlas-based analysis.

The construction of cortical surface was based on 3D T1-images using FreeSurfer version 5.3 (http://surfer.nmr.mgh.harvard.edu). The study workflows of the analyses are depicted in Fig. 1. The detailed procedure for surface reconstruction has been described and validated in previous studies^19^. In short, surface reconstruction is performed in a step-by-step procedure; namely, first the multi-scale analysis, next computation of the curvature of patches of the surface, followed by surface deformation and numerical integration. In the multi-scale analysis, to make the detection of grey or white and pial boundaries less sensitive to noise, we constrain the surface representation to be smooth. For computing the curvature, we used a technique that produces a surface that is second-order smooth, i.e., has a continuous second derivative. The surface deformation is implemented by using gradient descent with momentum. The numerical integration was carried out at four decreasing scales for the smoothing kernel until the error function decreased by less than 1%. The cortical thickness is computed as the average distance measured from each surface to the reconstructed surface from our T1 images.

The *first analysis* aimed to identify the clusters on the whole brain, which correlate with better clinical outcome based on dUPDRS using vertex-by-vertex analyses. In order to pinpoint the exact topographic specificity of the analysed effects, we calculated a whole-brain, hemisphere-specific vertex-by-vertex analysis using a uni-variate general linear model for the correlation between dUPDRS and cortical thickness. The vertex-by-vertex analysis was done to prevent the surface from intersecting itself and involved a spatial information table in conjunction with fast triangle-triangle intersection code. The resulting computational algorithm was linear in the number of vertices in the surface representation. Specifically, if the vertex results in an intersection, the size of that vertex is reduced until the self-intersection no longer occurs. The entire procedure is carried out in a multiscale manner, with the target intensity calculation using derivative information computed from images smoothed with a Gaussian kernel of a 10th standard deviation. The numerical integration continues until the error function becomes asymptotic. The standard deviation of the smoothing kernel is then decreased, the target intensities are recomputed, and the integration is repeated until a predefined minimum scale is reached.

The goal of the *second analysis* was to identify the cortical regions which could act as predictors for each hemisphere separately based on the *DBS voltage*. To this end, we analysed each hemisphere separately in a further correlative analysis of the *DBS voltage* and cortical thickness. We used age and disease duration as nuisance factors. Statistical results were considered significant at p < 0.001, uncorrected. Conditional on a significant F value in the analysis of covariance (ANCOVA) analyses, we performed Bonferroni post-hoc corrections for multiple comparisons (p < 0.05; two-tailed). The ANCOVA is a general linear model that blends the ANOVA and regression. This analysis decomposes the variance in the dependent variables into the variance explained by the covariates and residual variance.

The *third analysis* aimed to identify the interactions between the clinical outcome dUPDRS and the identified predictors (“cortex areas”) from the first analysis. To develop clinically feasible pathways at increased statistical power, we further analysed concatenated data from both hemispheres. We utilized Freesurfer to automatically label and assign at the neuroanatomical level the regional surface based on the probabilistic information estimated from a manually labeled training set. Using both geometric information and derived cortical model information on regional cortical thickness, regional values were extracted and pooled for either hemisphere and included into the ANCOVA model using STATISTICA Statsoft software. Using an integrated atlas, we also calculated the cortical thickness values for the regions that showed significant effects. These were included into an ANCOVA for the clinical predictor “dUPDRS” and within-subject factor “*cortex areas*”.

## Results

The clinical outcome markedly improved after STN-DBS (UPDRS_III_ MED OFF/STIM ON 19.2 ± 9.4) in comparison to the preoperative MED OFF condition (UPDRS_III_ 38.87 ± 11.71, t = 7.2, p < 0.001) and was not significantly different from the preoperative MED ON (with medication) state (UPDRS_III_ 18.67 ± 8.15, p > 0.05). The pre- and postoperative medication OFF (with postoperative stimulation switched off, STIM OFF) conditions did not differ (postoperative UPDRS_III_ MED OFF/STIM OFF 40.2 ± 8.3, p > 0.05).

**Figure 2:**
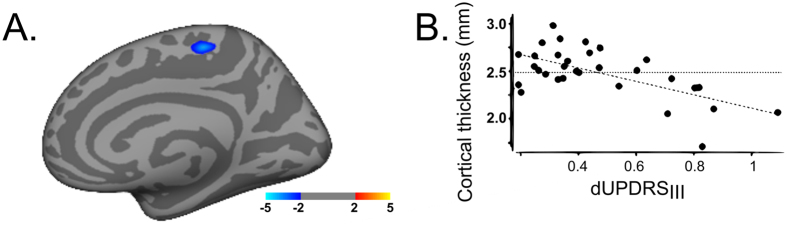
Cortical thickness changes and motor improvement. Cortical thickness correlation with the postoperative outcome as derived from the motor score improvement after DBS in comparison to preoperative state. Atrophy in the highlighted areas was linked to a poor motor postoperative outcome. Significant cluster at p < 0.001. Displayed images are presented at P < 0.05 threshold to better illustrate the anatomic extent of the area and the relative specificity of the findings. The colour bar shows the t-values. Correlation between motor improvement as shown by dUPDRS and cortical thickness (mm).

The *first* correlative analysis of clinical improvement after DBS and cortical thickness revealed clusters with significant interactions. A poor clinical outcome (as described by a high dUPDRS) was correlated with cortical thinning in the paracentral region (t_max_ = −3.77, Fig. 2A and B).

**Figure 3:**
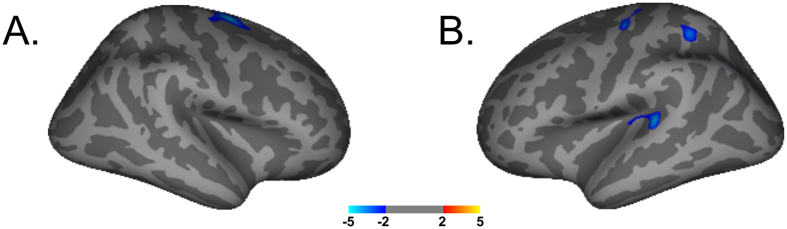
Cortical thickness changes and voltage correlation. Cortical thickness correlation with post-operative DBS voltage for an optimal clinical outcome. Significant clusters at P < 0.001. Displayed images are presented at p < 0.05 threshold to better illustrate the anatomic extent of the areas and the relative specificity of the findings. In the right hemisphere the superiorfrontal region and in the left hemisphere the superiorfrontal region, precuneus, superiortemporal, inferiorparietal and superiorparietal areas predicted an optimal postoperative outcome at low stimulation voltages. The colour bar shows the t-values.

The *second* analysis of the *DBS voltage* for optimal clinical response and cortical thickness revealed further significant interactions. An optimal clinical response at a low stimulation voltage was associated in the right hemisphere with an increased cortical thickness in the superiorfrontal region (t_max_ = −4.33, Fig. 3A). For the left hemisphere, the analysis revealed that the microstructural integrity in superiorfrontal region (t_max_ = −4.1), precuneus (t_max_ = −4.1), superiortemporal (t_max_ = −4.0), inferiorparietal (t_max_ = −3.4) and superiorparietal areas (t_max_ = −3.3) predicted an improved postoperative outcome at low stimulation voltages (Fig. 3B).

The *third* atlas-based analysis of the cortical thickness values from both hemispheres showed a significant dependency between the clinical improvement after DBS and cortical thickness in the frontal areas. The ANCOVA including the cortical thickness data from the regions indicated by the second analysis (paracentral, superiorfrontal, precuneus, superiortemporal, inferiorparietal and superiorparietal) and the continuous factor “dUPDRS” revealed a significant main effect for the factor *cortex area* [F(5, 300) = 45.8, p < 0.001]. The interaction between the factors *cortex area* and dUPDRS was also significant [F(5, 300) = 4.11, p < 0.001]. Post-hoc testing revealed that the clinical outcome correlated with cortical thickness values from the paracentral area (r = 0.35, F = 8.6, p = 0.005) and superior frontal region (r = 0.34, F = 8.25, p = 0.005). There were no significant effects for the other regions analysed.

## Discussion

Using anatomical 3D T1-images, we show that the integrity of the frontal cortex (paracentral area and superior frontal region), as derived from cortical thickness analysis, predicts the effects of STN-DBS in PD patients. Moreover, cortical thinning in these regions is not only associated with worse clinical outcome but also demands higher stimulation intensities for an optimal clinical response at a later stage.

We analysed the improvement in the motor scores and linked it to parameters of cortical integrity. STN-DBS markedly improved clinical symptoms. The UPDRS_III_ scores improved after STN-DBS showing a modulation of the motor aspects of the disease in the medication-free assessment. The clinical improvement after STN-DBS correlated with the cortical thickness values from the central and superior frontal areas suggesting a topographic specificity of the effects in the frontal cortex. Patients with atrophy of these areas had an inferior outcome to those with an intact cortical morphology.

Previous studies showed grey matter losses in sub-cortical structures such as thalamus, caudate and putamen in patients with PD^20^ while the cortical integrity remained preserved until later disease stages^21^. Voxel based morphometric (VBM) studies showed volumetric atrophy in fronto-temporal regions associated with PD^22^. In comparison to VBM studies, cortical thickness analyses might be more sensitive to grey matter integrity changes and reveal even more subtle atrophy patterns, and thus their use in clinical studies would be advantageous^23^. Cortical thinning early in the disease course was detected merely in PD patients with neurocognitive or executive impairment^15^,^24^. However, our analyses show that neurodegenerative processes mirrored in cortical thinning are pathophysiological relevant. A quantification of these patterns adds important information for therapy decisions.

These cortical regions are a main part of the motor network playing the essential role in movement generation and control^25^. Furthermore, the primary motor cortex and premotor cortical areas are a part of the motor inhibition network which is altered in PD patients^26^. These areas are closely interconnected through cortico-cortical U-fibers^27^ and possess strong connections to deeper sub-cortical regions.

These direct STN frontal cortex connections were shown to mainly contribute to the specific therapeutic effects of STN-DBS in rodents^10^. In humans, we see that the integrity of the frontal cortex, driven possibly by cross-talk through the hyperdirect pathway from the stimulation site in the STN, determines the outcome in patients. Since we have no information about the exact position of electrode placement, we cannot test its relation to the STN or to probability values of the hyperdirect pathway, with the motor outcome or necessary stimulation paradigm, but that should be addressed in future studies.

Furthermore, there is an urgent need in the clinical setting for the development of objective and investigator-independent automated algorithms that can accurately identify patients that would benefit from DBS therapy. Several clinical parameters have been previously analysed as predictors of the postoperative clinical outcome of STN-DBS. The only one that is clinically accepted derives from the observation that the lower the preoperative MED ON UPDRS_III_ motor score, the better the effects from STN-DBS, confirming that dopaminergic response is a predictor of postoperative outcome. The proposed atlas-based approach allows, however, an independent, automated and easy to use paradigm for the quantification of the atrophy metrics that can be used for outcome prediction in the clinical setting.

In conclusion, our data suggests that the effects of STN-DBS in PD depend on cortical microstructural pattern, which can be used to better elucidate the achieved systemic effects and predict the long term outcome.

## Additional Information

**How to cite this article**: Muthuraman, M. *et al*. Effects of DBS in parkinsonian patients depend on the structural integrity of frontal cortex. *Sci. Rep.* **7**, 43571; doi: 10.1038/srep43571 (2017).

**Publisher's note:** Springer Nature remains neutral with regard to jurisdictional claims in published maps and institutional affiliations.

## Figures and Tables

**Table 1:** Demographic details of patients.

n	31
Male/Female	23/8
Age	63.4 ± 9.3
Disease duration (years)	16.0 ± 6.2
Hoehn and Yahr scale (medication ON/OFF)	2.6 ± 0.7/3.5 ± 0.8
Preoperative UPDRS_III_ scores (medication ON/OFF)	18.67 ± 8.15/38.87 ± 11.7

Values reported as mean ± std.
